# Myokine Irisin promotes osteogenesis by activating BMP/SMAD signaling via αV integrin and regulates bone mass in mice

**DOI:** 10.7150/ijbs.63505

**Published:** 2022-01-01

**Authors:** Yuan Xue, Sihan Hu, Chichi Chen, Jiachen He, Jie Sun, Yesheng Jin, Yuanshu Zhang, Guoqing Zhu, Qin Shi, Yongjun Rui

**Affiliations:** 1 Department of Orthopedics, the First Affiliated Hospital of Soochow University, Orthopedics Institute of Soochow University, Medical College of Soochow University, Suzhou, Jiangsu, 215006, P. R. China.; 2 Department of Orthopedics, Wuxi Ninth People's Hospital affiliated to Soochow University, Wuxi, Jiangsu, 214026, P. R. China.; 3 Department of Physiology, Nanjing Medical University, 101 Longmian Avenue, Nanjing, Jiangsu, 211166, P. R. China.

**Keywords:** Irisin, mesenchymal stem/stromal cell, Osteogenesis, BMP/SMAD signaling, αV integrin.

## Abstract

Irisin is well-known to contribute to bone homeostasis due to its bidirectional regulation on osteogenesis and osteoclastogenesis. However, the mechanisms of irisin involved in mesenchymal stem/stromal cells (MSCs)-derived osteogenesis are still under investigated. Fibronectin type III domain-containing protein 5 (FNDC5) is the precursor protein of irisin, compare with wild type (WT) littermates, FNDC5^-/-^ mice lost bone mass significantly, collectively evidenced by the decrease of bone mineral density (BMD), impaired bone formation and reduced N-terminal propertied of type I procollagen (P1NP) in sera. Meanwhile, the bone resorbing of FNDC5^-/-^ mice has enhanced accompanied by increased tartrate phosphatase (TRAP) staining cells morphologically and cross-Linked C-telopeptide of type 1 collagen (CTX) level in sera. *In vitro* study showed that lack of irisin impeded the MSC-derived osteogenesis of FNDC5^-/-^ mice. The addition of irisin promote the osteogenesis of WT and irisin-deficient MSCs, by activating αV integrin-induced ERK/STAT pathway**,** subsequently enhancing bone morphogenetic protein 2 (BMP2) expression and BMP/SMAD signaling activation. Taken together, these findings further indicate that irisin regulates bone homeostasis. Moreover, irisin promotes MSC-derived osteogenesis by binding to αV integrin and activating BMP/SMAD signaling consequently. Thus, irisin may be a promising therapeutic target for osteoporosis and bone defects.

## Introduction

Osteoporosis has become one of the most significant public health issues characterized by the reduction of BMD with the increased probability of fracture 1. In China, 70%-80% of fractures in the elderly were caused by osteoporosis 2. With the life expectancy of human and the aging of world population trend intensifying, this problem continued to deteriorate 3. By their very nature, osteoporosis is caused by excessive bone loss. In terms of incentives. New bone formed by osteoblasts is less than old bone absorbed by osteoclasts, which destroys the homeostasis of bone metabolism and produces a negative balance effect. There are a class of drugs that quite effective in the treatment of osteoporosis in recent years 4. Currently, the anti-osteoporosis drugs wildly used in clinic including estradiol for preserve postmenopausal osteoporosis (PMOP) 5, calcitonin gene-related peptide (CGRP) in osteoporosis patients 6, bisphosphonates by inhibiting bone resorption 7. Denosumab is first used to inhibit receptor activator of nuclear factor-kB ligand (RANKL), which combined with its receptor RANK, induces osteoclast precursors mature into functional osteoclasts 8, 9. In a nutshell, the current treatment of osteoporosis is more focused on the precise treatment of osteogenic differentiation and osteoclast activation. Therefore, maintaining bone metabolic homeostasis by regulating the subtle metabolic processes of osteoblasts and osteoclasts has still been a hotspot in osteoporosis research.

Osteoblasts are mainly derived from MSCs. Upon inductive conditions, MSCs may differentiate into osteoblasts, chondrocytes, myoblasts, adipocytes, and other functional cells 10. Therefore, MSCs are considered as promising multipotent stem cells especially as the resource of osteoblasts and widely applied in the bone metabolic diseases and tissue engineering 11, 12. Multiple regulations including biological, physical, and chemical factors control osteogenic differentiation of MSCs 13. Aging and metabolism as the most important elements will reduce the number of MSCs and the ability of osteogenic differentiation, leading to abnormal differentiation of MSCs 14. Taken together, it has been a focus of enhancing not only MSCs proliferation but also MSCs differentiation towards the osteoblastic fate.

Activation of osteoclast is another important part in bone remodeling 15. It originates from hematopoietic stem cells (HSCs), degrades bone by secreting acids and proteolytic enzymes such as cathepsin K (CTSK) after activation, then dissolves collagen and other matrix proteins produced during bone resorption to absorb old and damaged bones 16. In sum of, bone remodeling begins with the absorption of bone matrix by osteoclasts, followed by the recruitment of osteoblast-related cells to the resorption site for remodeling, adapting to changes in the mechanical environment of the body or repairing bone injury through the formation and secretion of bone matrix 17.

Currently, skeletal muscle is identified as endocrine organ secreting sorts of myokines such as myostatin, IL-6, insulin. These myokines act on lots of organs and tissues such as adipose, liver and bones 18. As a new type of muscle factor secreted during exercise, irisin was discovered by Bostrom et al. in 2012. It has been demonstrated that exercise could increase peroxisome proliferator-activated receptor-gamma coactivator-1α (PGC1α), upregulate the expression of membrane protein FNDC5, promote the release of its cracking product irisin, and finally facilitate the browning of white adipose tissue to regulate energy metabolism 19. It is mentioned above that, the transformation of MSCs into adipocytes and osteoblasts has a special relationship with the pathological environment of abnormal bone remodeling 13. Therefore, in addition to its importance in lipid metabolism, the effect on bone metabolism has also been confirmed by lots of research. Studies have shown that the level of irisin was negatively correlated with fragile vertebral fractures and had no significant correlation with bone density and obesity 20. Qiao et al. has reported that osteoblast differentiation was mediated by activating p38 mitogen-activated protein kinase (MAPK) and extracellular signal-regulated kinase (ERK), irisin directly act on osteoblast, promoting osteoblast both proliferation and differentiation *in vitro* 21. Meanwhile, irisin could reduce the number of osteoclasts and increase the quality and strength of cortical bone 22. These studies demonstrated that irisin contained a certain positive effect on bone metabolic balance and could be a great potential application in osteoporosis.

It was reported that in RAW264.7 macrophages, irisin inhibited inflammatory cytokine generation and M1 macrophage polarization induced by lipopolysaccharide (LPS) via Adenosine Monophosphate Activated Protein Kinase (AMPK) phosphorylation 23. Another study indicated that FNDC5/irisin was required for ovariectomy-induced osteoporosis and endogenous FNDC5/irisin induced bone resorption, partly through its application to osteocytes 24. FNDC5 conditional deficiency in osteoblast has been used as a loss-of-function model to determine the effects of irisin deficiency during bone development and physical activity 25. However, further exploration of irisin in the whole process of bone formation and development and its possible new mechanism of action are still lacking.

Therefore, in our present study, FNDC5^-/-^ and littermate WT mice were used to evaluate the bone mass during the growth period. We found that lack of FNDC5/irisin could accelerate the progress of osteoporosis, and demonstrated that irisin regulated ERK/STAT3 signaling pathway through binding to membrane receptor integrin αV, and further affected bone metabolism BMP/SMAD pathway to promote osteogenic differentiation of MSCs during the *vitro* osteogenic induction. Through this study, we further verified the important role of irisin in osteoporosis and explored possible new regulatory pathways to expand the research direction of irisin in bone metabolism and provide a possible new target for the treatment of osteoporosis.

## Materials and Methods

### Animals

The experimental protocols of the mice were followed the National Institute of Health's Guide for the Care and Use of Laboratory Animals and permitted by the Animal Care and Use Committee of Soochow University. Global deletion of the FNDC5 gene in mice (FNDC5^-/-^) were generously provided by Department of Physiology, Nanjing Medical University (Nanjing, Jiangsu, China). WT and FNDC5^-/-^ mice global deletion of the FNDC5 gene in mice (FNDC5^-/-^) were on a C57BL/6 background, and the identification was performed in accordance with the literature 26, while the genotype identification was placed in supplementary Figure 1. All mice were fed in the specific pathogen-free (SPF) animal facility at Laboratory Animal Center of Soochow University. FNDC5^-/-^ mice and WT littermates were sacrificed at postnatal 6, 12, 18 and 24weeks, Tissues and sera were collected immediately and stored at 4 °C.

### Micro-computed tomography (micro-CT) and Analysis

The distal femoral epiphyses were scanned using the micro-CT system (Skyscan 1176, Bruker, Belgium). Bones were scanned at a high resolution (9μm) with an energy of 50 kV, 200 mA of intensity and regular increments of 0.7 in the 180° rotational steps. Three-dimensional (3D) reconstruction of CT scan data and analysis of bone related parameters: BMD, bone volume/tissue volume (BV/TV), trabecular thickness (Tb.Th), trabecular number (Tb.N), trabecular separation (Tb.Sp) were performed according to previous research methods 12.

### Enzyme Linked Immunosorbent Assay (ELISA)

In this study, P1NP and CTX in the sera of the mice were analyzed by ELISA kits: (Elabscience, China), according to the instructions of manufacturer.

### Histological and immunological staining

The femurs were decalcified in 14% ethylene diamine tetraacetic acid (EDTA) for 28 days. Both frozen sections and paraffin-embedded sections were prepared. Hematoxylin and eosin-staining (H&E-staining), TRAP staining (Sigma Aldrich, USA) for osteoclast, masson trichrome staining (Salorbio, G1340, China) for new immature collagen was performed histologically. Frozen sections were used for immunohistochemical staining with antibodies to analyze the expression of osteogenetic markers: Phosphorylated-Smad1/5/9 (CST, USA), Osterix (Abcam, USA).

### Cell culture

Isolating and culturing bone marrow MSCs (BMSCs) and bone marrow macrophages cells (BMMs) were followed the methods described in the previous articles 12, 27. The osteogenesis induction medium was supplemented with dexamethasone (Dex) at a concentration of 1x10^-9^M/L, 1% β-glycerophosphate and 0.2% AA. 50ng/ml RANKL (R&D, USA) and 30 ng/ml macrophage colony stimulating factor (M-CSF) (R&D, USA) were used for osteoclastgenesis of BMMs, then we did osteoclast staining by TRAP kit and examined under a microscope (Zeiss, Germany) after 6 day-induction.

### Cell Counting Kit-8 (CCK-8)

BMSCs were inoculated into 96-well plates with 2X10^3^/well and added with different concentrations of recombinant irisin (0.1 ng/ml, 1 ng/ml, 5 ng/ml, 10 ng/ mL, 20 ng/ mL). Cell proliferation was detected by cell counting kit at day 1, 3, 5. The medium was removed at the same time point, and the medium containing 10% CCK-8 liquid was added, then incubated in an incubator for 2 hours without light. At last, the medium was transferred to a new well plate and the absorbance was read at 450 nm.

### Colony formation units

All bone marrow primitive cells of WT and FNDC5^-/-^ mice were collected from bone marrow flushing. Then cells were directly seeded in the well plate at a rate of 3 million/well and cultured in the medium containing 10% fetal bovine serum (FBS). After culturing for one week, the culture medium was sucked out, and washed twice with PBS, fixed by 4% paraformaldehyde for 15 minutes. After washing with PBS, the crystal violet solution was added for staining. The efficiency of cell clone formation was analyzed according to the number and size of purple nodules.

### Alkaline Phosphatase (ALP/AKP) Staining and AKP Activity

On the seventh day of osteogenesis induction, BMSCs were fixed by 4% paraformaldehyde for 30 minutes, then covered with BCIP/NBT working solution (Beyotime, China) for 20 minutes in dark. The cells were observed under a microscope and photographed. After the protein concentration was measured by BCA protein quantitative kit, AKP staining kit (Jiancheng, China) was used to measure the OD values of blank, standard and measured wells by spectrophotometer at 520 nm, and AKP activity was calculated by formula.

### Alizarin Red S (ARS) Staining and Quantification

After osteogenic induction for two weeks, BMSC-derived cells were fixed with 4% paraformaldehyde. ARS staining solution (Cyagen, China) was used to stain for 20min. Finally, ddH_2_O was used to wash for three times. The mineralized nodules containing calcium stained in red and images were captured by microscope. The quantification of ARS staining was carried out in accordance with previous studies in our laboratory 12. 5% perchloric acid was added to dissolve the calcium nodules. After 30 minutes incubation, detected the absorbance (optical density) at 490 nm.

### Quantitative real-time PCR (qRT-PCR)

Extract the total RNA with Trizol (Beyotime, China). NanoDrop-2000 (Thermo Fisher, USA) was used to survey the density. Reverse transcriptase reactions were done by Prime Script RT reagent Kits (TaKaRa, Japan). SYBR Premix Ex Taq™ (BIO-RAD, USA) was used to qRT-PCR. GAPDH was used as internal reference, and the quantitative results were calculated by 2 ^ΔΔCT^ method. The sequences of mouse primer were listed in Table 1.

### Western Blot

Cells were resuspended with RIPA buffer (NCM Biotech, China) and total protein was collected. BCA kit (Beyotime, China) was used to detect the concentration of protein sample. After electrophoresis and membrane transfer processes 27, the fiber membranes were incubated with primary antibodies against Smad1/5/9, BMP2, Smad4, phospho-STAT3, BMPR2, Erk1/2, Integrin αV, runt-related transcription factor 2 (RUNX2), osteopontin (OPN)(Abcam, ab66737, ab96826, ab40759, ab76315, ab130206, ab17942, ab179475, ab236639, ab283656, USA), phospho-Smad1/5/9 (CST, 13820S, USA), β-Actin (Beyotime, AA128, China) respectively, then they were incubated with corresponding secondary antibodies. In the end, the target bands were developed with chemiluminescence reagent (Thermo Fisher Scientific, USA) and imaged under ChemiDoc Touch Imaging System (Bio-Rad). Quantification of the band intensity was performed using Image J software.

### Statistical Analysis

Data were analyzed by SPSS 19.0 software (SPSS Inc, Chicago, IL, USA) and presented as mean ± standard deviation from at least three independent samples. Paired *t*-test was used to compare two groups, and one-way analysis of variance was used to compare more than two groups. *P* < 0.05 was considered as a significant difference.

## Results

### FNDC5 deficiency in mice reduced bone mass

**Figure 1:**
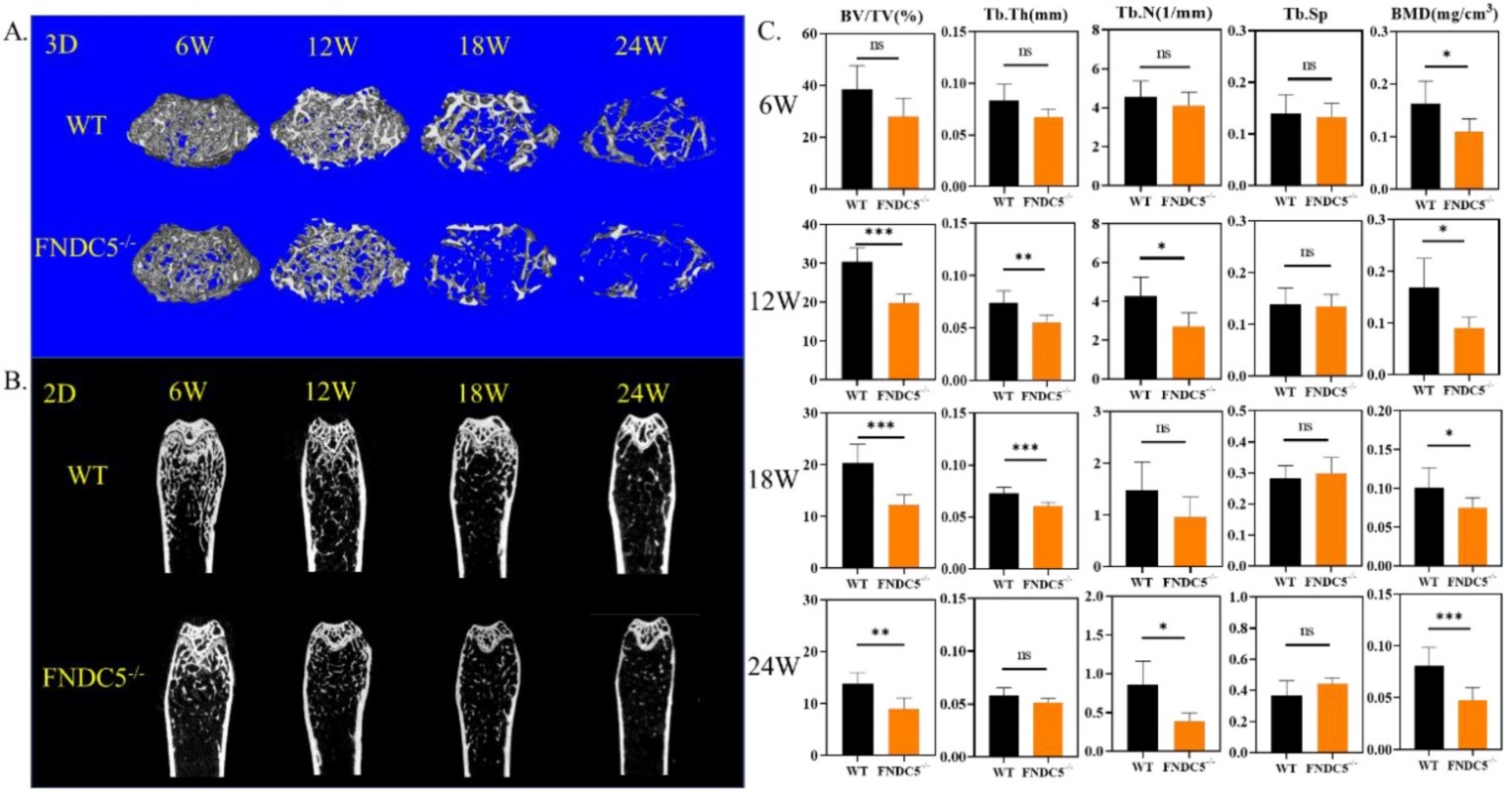
FNDC5 deficiency in mice reduced bone mass. (A) 3D and (B) 2D reconstruction of micro-CT images of femurs. (C) Bone parameters of femurs at indicative time point. BV/TV (%), Tb.Th (mm), Tb.N (1/mm), Tb.Sp, BMD (mg/cm^3^), (n=6, *P < 0.05, **P <0.01, ***P <0.001).

Since fibronectin type III domain-containing protein 5 (FNDC5) is the precursor protein of irisin, FNDC5 deficiency mice are lack of irisin. We detected bone mass of FNDC5 deficiency mice at different growth points. Micro-CT images showed that, compared with littermate WT mice by 3D&2D-rebuilt, FNDC5^-/-^ mice showed no significant difference in bone mass during the first 6 weeks. However, from the 12th week, the cancellous bone mass of FNDC5^-/-^ mice decreased significantly (Figure 1A-B). The quantitative analysis of bone parameters accurately revealed that the BV/TV, Tb.Th, Tb.N, and BMD of FNDC5^-/-^ mice were reduced by one third compared with WT mice at the indicated time points (Figure 1C). Herein, the lack of FNDC5/irisin affect the bone development process of mice.

### FNDC5 deficiency in mice reduced bone formation and increased bone resorption

**Figure 2:**
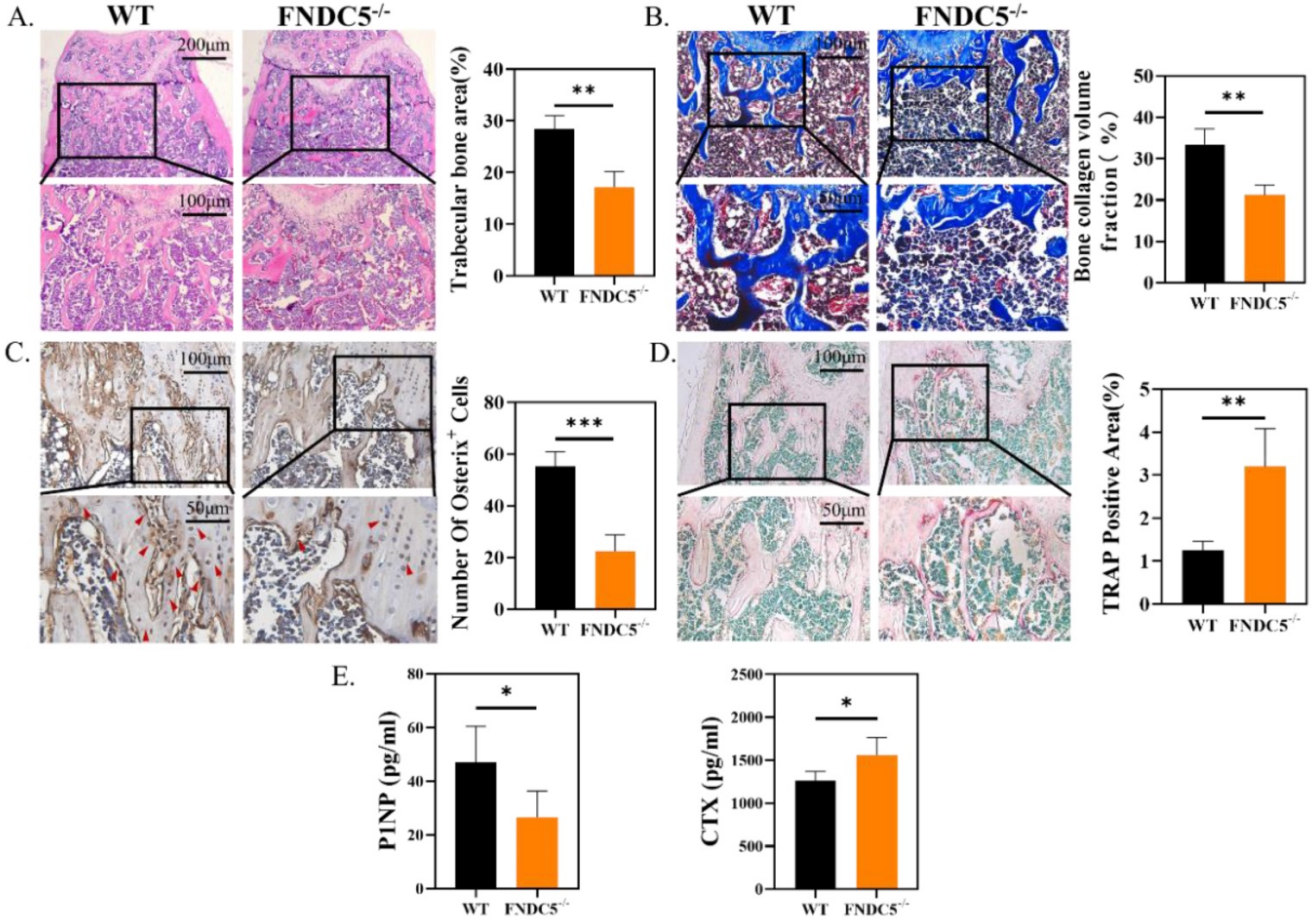
FNDC5 deficiency in mice reduced bone formation and increased bone resorption. (A) H&E-staining of femur section. (B) Masson trichrome staining showing new bone collagen fibers in blue and red for mature bone. (C) Immunohistochemical staining indicating Osterix expression level in femur. (D) TRAP staining of femur sections. (E) P1NP, CTX in serum determined by ELISA. (n=6, *P < 0.05, **P <0.01, ***P <0.001).

Consistent with the results of micro-CT, histological analysis confirmed the protective effect of irisin in FNDC5^-/-^ mice. Obviously, the absence of FNDC5 resulted in thinner and disordered trabeculae under the epiphyseal plate compared with the ones of WT group (Figure 2A). In Masson staining, bone collagen fibers (in blue) was reduced about 36% below the femoral metaphysis of FNDC5^-/-^ mice (Figure 2B). Immunohistochemical staining of the metaphysis of the femur in mice displayed that the number of Osterix positive cells in WT mice was nearly three times higher than that in FNDC5^-/-^ mice (Figure 2C). The results further indicated that TRAP positive area on the bone trabecular surface in FNDC5^-/-^ mice was more than three times as large as that in WT mice (Figure 2D). As expected, the bone formation marker P1NP dramatically decreased and the bone resorption marker CTX increased in the sera of the 24-week-old FNDC5^-/-^ mice (Figure 2E). Collectively, deficiency of FNDC5/irisin reduced bone formation and promoted bone resorption, which eventually led to abnormal bone development in mice.

### Recombination irisin (r-irisin) promoted MSC-differentiated osteogenesis *in vitro*

**Figure 3:**
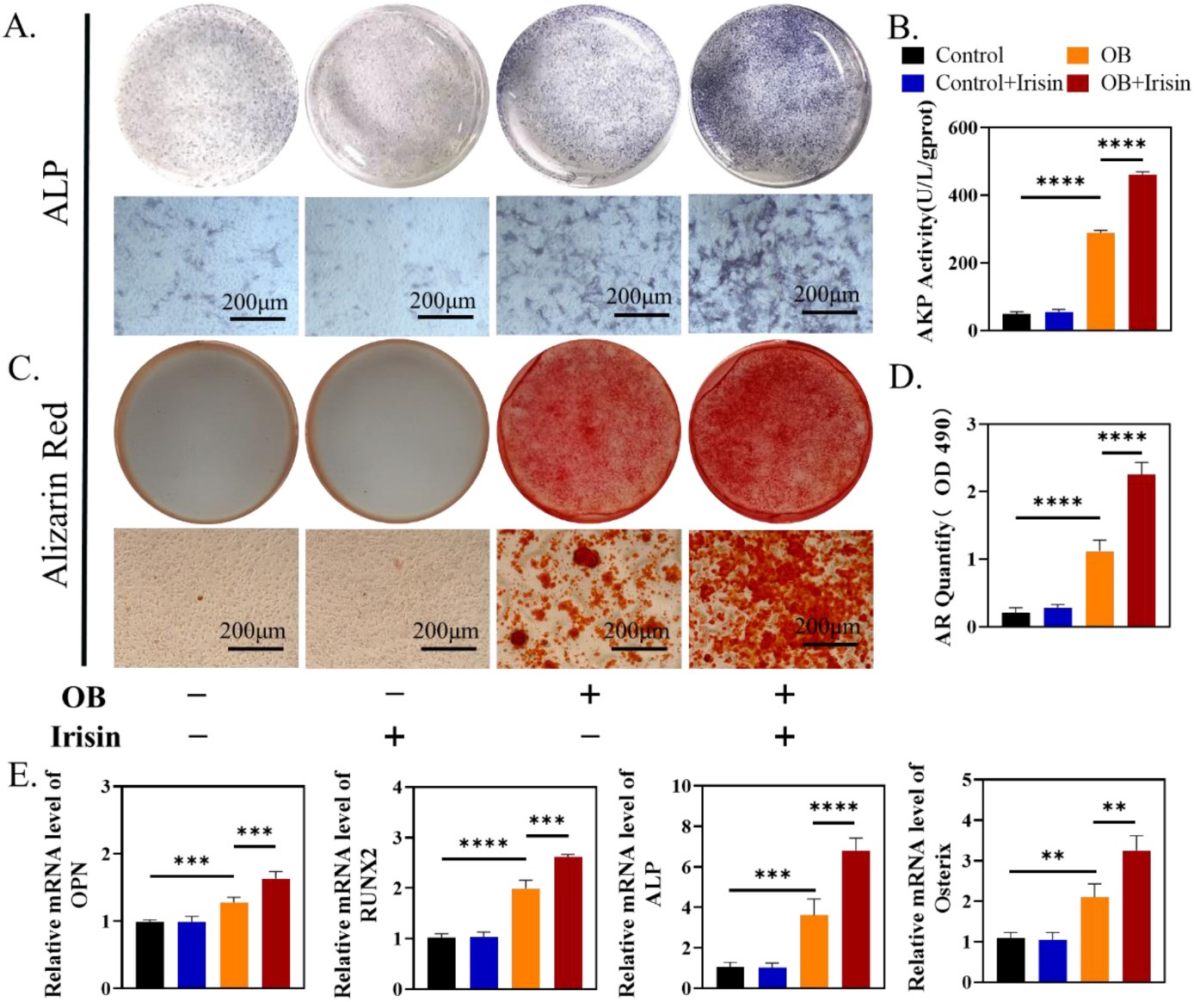
R-irisin promoted osteogenic differentiation *in vitro*. (A) ALP staining of BMSCs after osteogenic induction. (B) AKP activity. (C) Alizarin Red staining. (D) Semi-quantitative analysis of mineralized nodules. (E) qRT-PCR analysis of OPN, RUNX2, ALP, and Osterix after osteogenic induction for 7 days. (n=5, **P <0.01, ***P <0.001, ****P <0.0001).

To test if irisin facilitated osteogenesis directly, we treated mouse BMSCs with exogenous irisin *in vitro*. At the beginning, we explored the effect of r-irisin on BMSCs proliferation in a metrological gradient by CCK-8. The results indicated that, from 0.1 to 20ng/mL, r-irisin did not affect the BMSC proliferation (Supplementary Figure 2A). Live-death staining on the third day under the same condition showed that no dead cells appeared, further confirming the similar result (Supplementary Figure 2B-C). We chose r-irisin at a concentration of 5 ng/ml in the subsequent experiments to better simulate the effect of irisin on osteogenesis under physiological state^28^. After 7 days osteogenic induction of BMSCs, ALP staining was enhanced obviously by r-irisin (Figure 3A-B). ARS staining indicated that r-irisin also promoted the formation of mineralized nodules after 14 days osteogenic induction. Through quantitative analysis, it was clear that the ability to osteogenic differentiation of BMSCs was doubled after the addition of irisin (Figure 3C-D). Quantitative analysis of gene profiles showed that OPN, ALP, Osterix, and RUNX2 of BMSCs after induction were significantly higher when treated with r-irisin (Figure 3E). The Western blot results displayed the same trend as qRT-PCR (Supplementary Figure 3A-B). As mentioned above, it is an art-of-state that exogenous r-irisin could promoted osteoblast differentiation.

### Irisin deficiency did not affect the clonal formation efficiency of bone marrow cells but inhibited osteogenic differentiation

**Figure 4:**
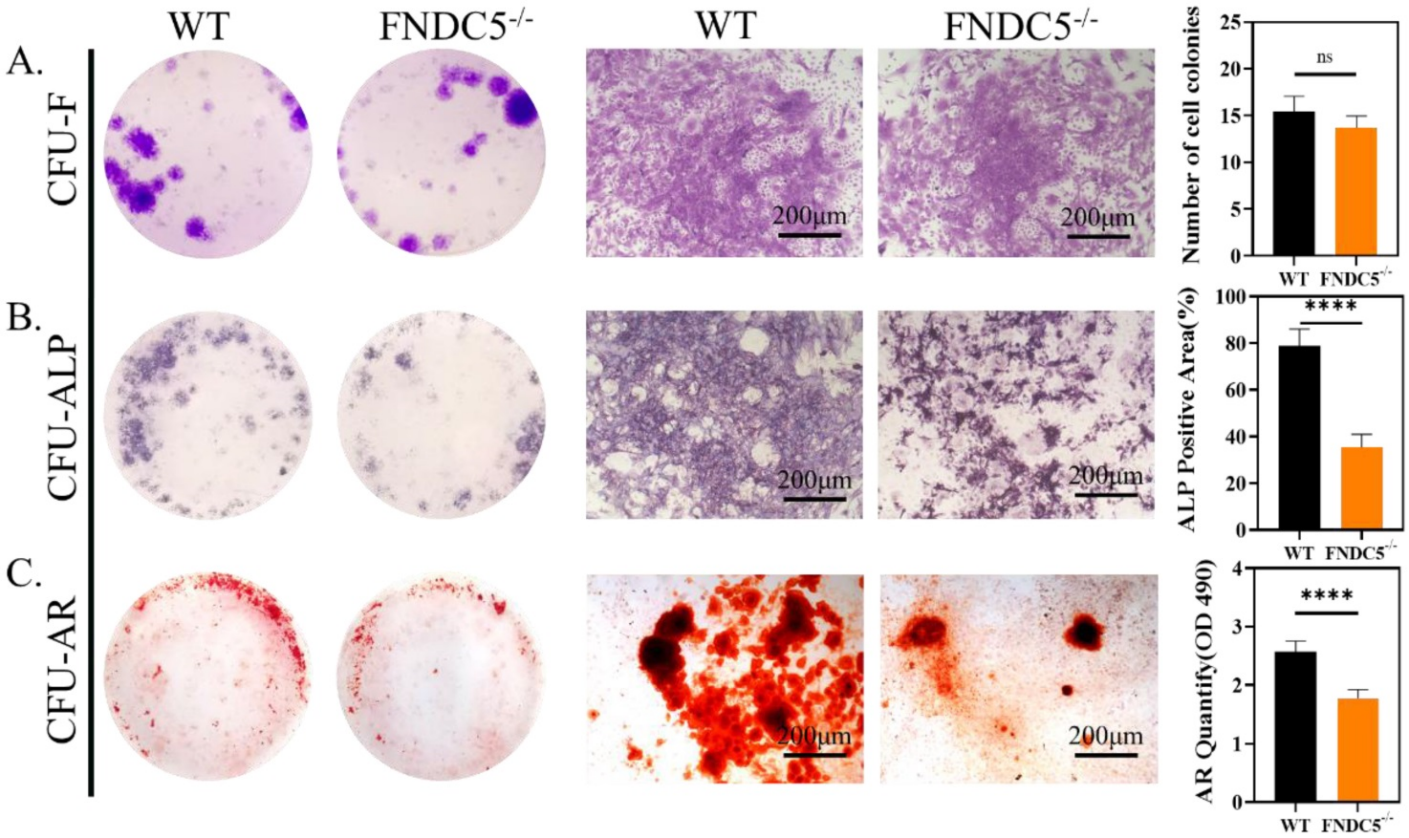
The clonal formation efficiency and osteogenic differentiation of bone marrow cells in WT and irisin deficiency mice. (A) Crystal violet staining and the number of cell colonies analysis after 7 days culture. (B) ALP staining and ALP positive area analysis after 7 days osteogenesis. (C) ARS staining and semi-quantitative analysis after 14 days osteogenesis (n=5, ****P <0.0001).

We collected bone marrow cells from WT and FNDC5^-/-^ mice at the age of 12 weeks, the cell colony formation units (CFU) were observed after direct cultured for 7days. The crystal violet staining showed no significant difference in the number of colonies between the two sources of cells (Figure 4A). After 7 days of osteogenesis, the CFU-ALP expression of BMSCs from WT mice was significantly higher than that of FNDC5-deficient mice (Figure 4B). The same result was also found in CFU-AR staining after 14 days of osteogenesis (Figure 4C). It was indicated that FNDC5/irisin deficiency did not affect the clonal formation efficiency of BMSCs but inhibited their osteogenic differentiation.

### R-irisin rescued the osteogenic differentiation reduction of BMSCs caused by FNDC5 deficiency

**Figure 5:**
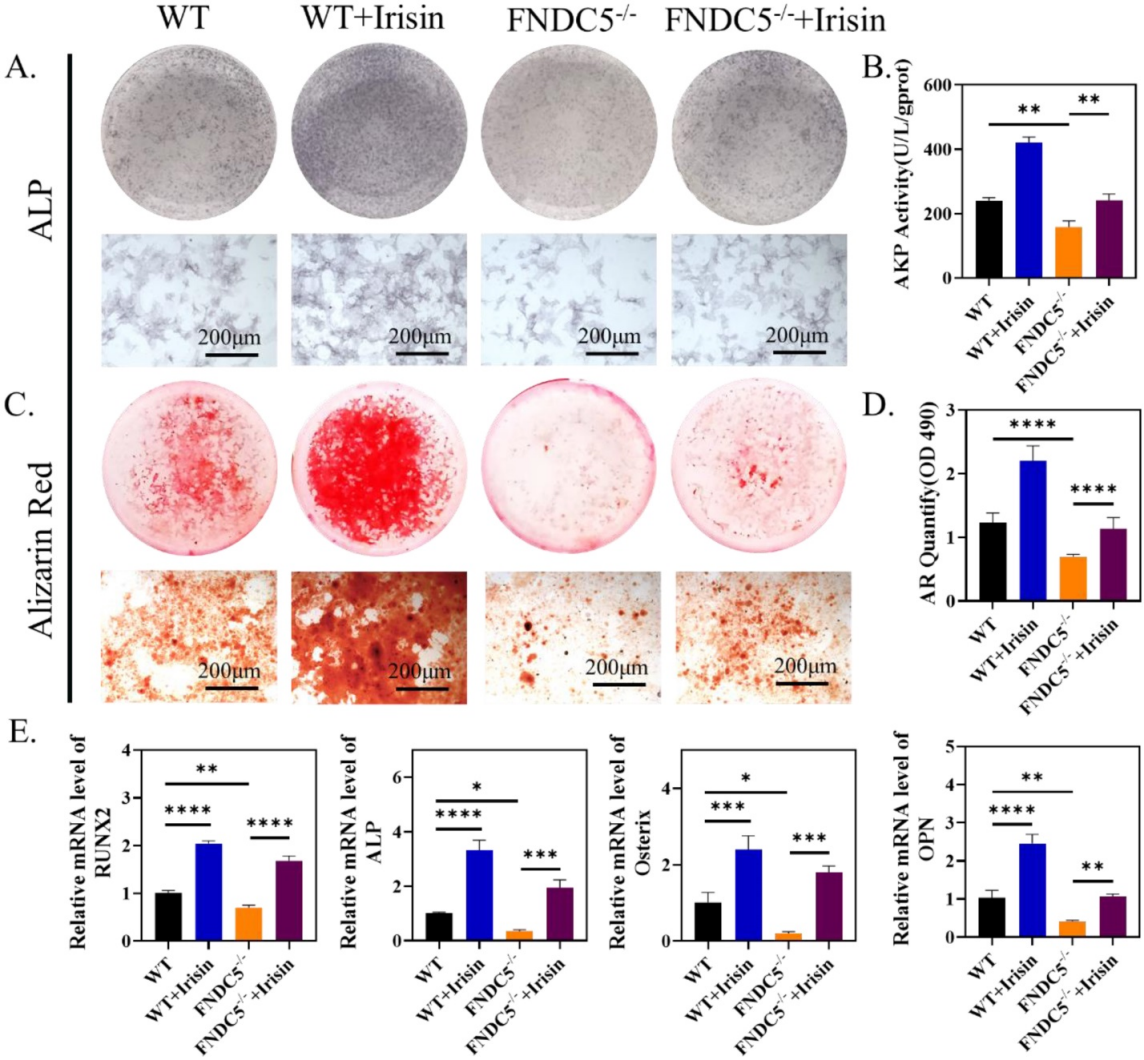
R-irisin rescued the reduced osteogenic differentiation of BMSCs caused by the deficiency of FNDC5. (A) ALP staining after osteogenic induction for 7 days. (B) AKP activity. (C) ARS staining after osteogenic induction for 14 days. (D) Quantitative analysis of ARS staining. (E) qRT-PCR analysis of the relative messenger RNA (mRNA) expression of the osteogenesis-related genes, including RUNX2, ALP, Osterix and OPN after osteogenic induction for 7 days (n=5, *P <0.05, **P <0.01, ***P <0.001, ****P <0.0001).

According to the previous micro-CT analysis, FNDC5-deficient mice showed significant bone mass loss at the age of 12 weeks (Figure 1A-C). Therefore, 12-week FNDC5^-/-^ and littermate WT mice were selected to isolate bone marrow BMSCs for osteogenesis induction and intervene with r-irisin. ALP staining showed about 28% reduction in FNDC5-deficient group (Figure 5A-B). ARS staining on the 14th day of induction displayed a 30% reduction in the number of mineralized nodules formed in the FNDC5-deficient group (Figure 5C-D). The analysis of qRT-PCR showed decreased expression of osteoblast-related genes in BMSCs from FNDC5^-/-^ mice after 7 days of osteogenic differentiation, including RUNX2, ALP, Osterix, OPN (Figure 5E). However, when FNDC5-deficient group added with r-irisin, the osteogenic ability reduction of BMSCs was rescued (Figure 5A-E). As expected, the results of Western blot showed the similar trend to qRT-PCR (Supplementary Figure 4A-B). In summary, r-irisin could rescue the osteogenic differentiation of BMSCs caused by FNDC5 deficiency.

### Both of endogenous and exogenous r-irisin restrained osteoclast activation

**Figure 6:**
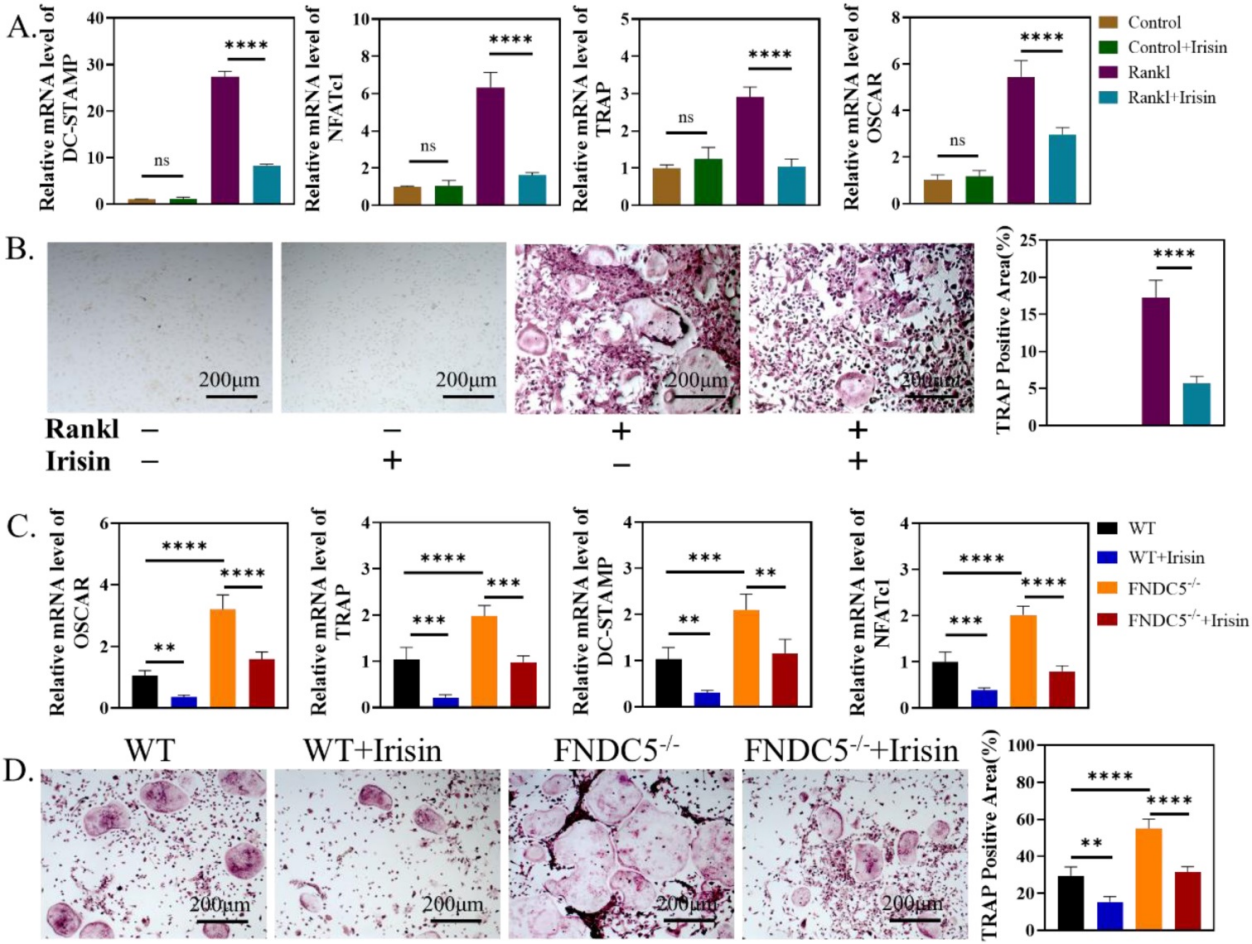
Endogenous and exogenous irisin restrain osteoclast activation. (A) qRT-PCR analysis of DC-STAMP, NFATc1, TRAP and OSCAR of WT mice after induction for 3 days. (B) TRAP staining of BMMs from WT mice after induction for 6 days. (C) qRT-PCR analysis of OSCAR, TRAP, DC-STAMP, and NFATc1 of BMMs from WT and FNDC5^-/-^ mice after osteoclastogenesis induction under the intervention of r-irisin for 3 days. (D) TRAP staining (**P <0.01, ***P <0.001, ****P <0.0001).

We further study the influence of irisin on the activation process of osteoclasts. First, r-irisin with the concentration of 5ng/ml was added to BMMs of WT mice in the process of osteoclast differentiation. On the third day, the expressions of osteoclast-related genes: dendritic cell-specific transmembrane protein (DC-STAMP), nuclear factor of activated T-cells c1 (NFATC1), TRAP and osteoclast-associated receptor (OSCAR) were significantly reduced (Figure 6A). TRAP staining and quantitative analysis on the 6th day showed that r-irisin could inhibit RANKL-induced osteoclast activation (Figure 6B). Then BMMs were extracted from WT mice and FNDC5^-/-^ mice aged 12 weeks for osteoclast induction, qRT-PCR analysis confirmed that during the process of osteoclast differentiation, the expression of osteoclast differentiation related genes: OSCAR, TRAP, DC-STAMP, and NFATc1 in BMMs of FNDC5^-/-^ mice were significantly up-regulated (Figure 6C), and more osteoclasts could be obtained (Figure 5D). However, these increases were obviously inhibited by the addition of r-irisin (Figure 5C-D). It is indicated that FNDC5/irisin deficiency could enhance osteoclast activation, while exogenous irisin was able to inhibit this phenomenon.

### Irisin promoted the phosphorylation of ERK/STAT through integrin receptor αV to up-regulate the expression of BMP2 and promote osteogenic differentiation of BMSCs

**Figure 7:**
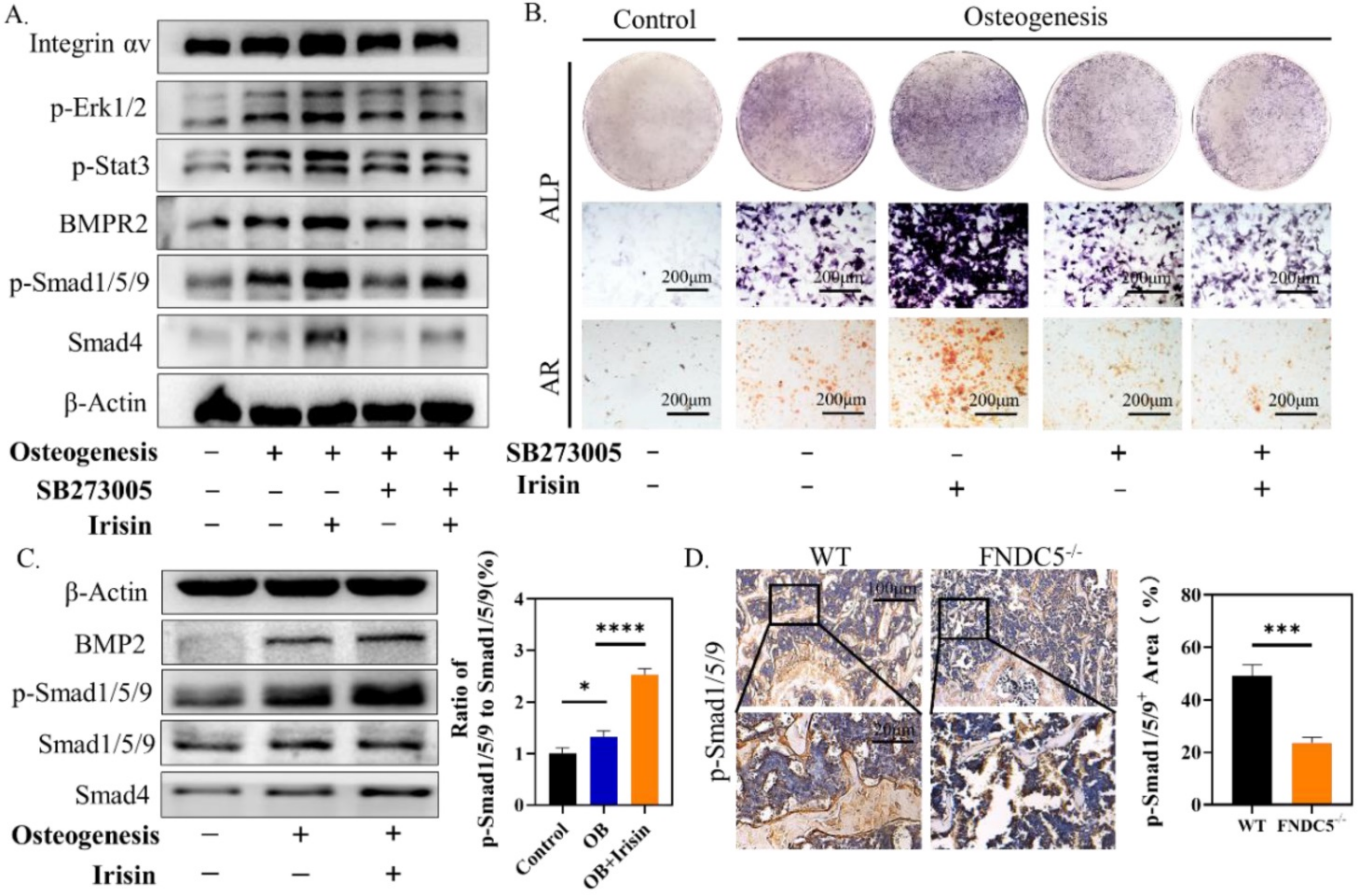
Irisin promoted the phosphorylation of ERK/STAT through binding to integrin receptor αV and up-regulates the expression of BMP2 to enhance osteogenic differentiation. (A) Cell lysates were subjected to immunoblotting analysis. Protein expression levels of integrin αV, p-Erk1/2, p-STAT3, BMPR2, p-Smad1/5/9, and Smad4 of BMSCs induced into osteoblasts for 3 days in the presence of r-irisin and SB273005. (B) ALP and ARS staining. (C) Protein expression levels of BMP2, Smad1/5/9, p-Smad1/5/9 and Smad4 after 3 days of osteogenic induction of BMSCs treated with r-irisin, and the ratio of p-Smad1/5/9 to Smad1/5/9 of the protein grayscale values. (D) Immunohistochemical staining of p-Smad1/5/9 in the femur of WT and FNDC5^-/-^ mice (n=6, *P <0.05, ***P <0.001, ****P <0.0001).

Studies have proved that STAT/BMP signaling pathway play a crucial role in the process of bone formation and development 29. To investigate the regulatory mechanism of irisin in osteogenesis, several signaling proteins were detected. SB273005, the inhibitor of αV integrin, and r-irisin were used to intervene the osteogenesis induction of BMSCs. While the expression of p-ERK1/2, p-STAT3, BMPR2, p-Smad1/5/9, and Smad4 were increased after r-irisin intervention, it was significantly reduced by addition of SB273005 (Figure 7A). The quantitative analysis of the western blot was shown in Supplementary Figure 5A. The ALP and ARS staining showed the same trend (Figure 7B). The BMP/SMAD signaling has been demonstrated as a crucial part in the process of bone formation and development. We detected BMP/SMAD signaling in the process of r-irisin-induced osteogenesis by Western Blot. During the osteogenic differentiation of BMSCs, after 3 days of r-irisin treatment, the protein expressions of BMP2, and Smad4 were increased (Supplementary Figure 5B). The ratio of quantitative analysis of phosphorylated Smad1/5/9 to non-phosphorylated in the irisin intervention group was doubled (Figure 7C). Immunohistochemical staining revealed that the expression of p-Smad1/5/9 in femur of FNDC5^-/-^ mice was reduced by 53% (Figure 7D). These results demonstrated that r-irisin affected the osteogenesis of BMSCs through the integrin αV receptor. At the same time, we speculated that irisin could activate the ERK/STAT signaling pathway through binding to the integrin αV receptor to increase the production of BMP2, and then combine with the membrane receptor BMPR2 to activate BMP/SMAD signaling to promote osteogenic differentiation at last.

## Discussion

**Scheme 1:**
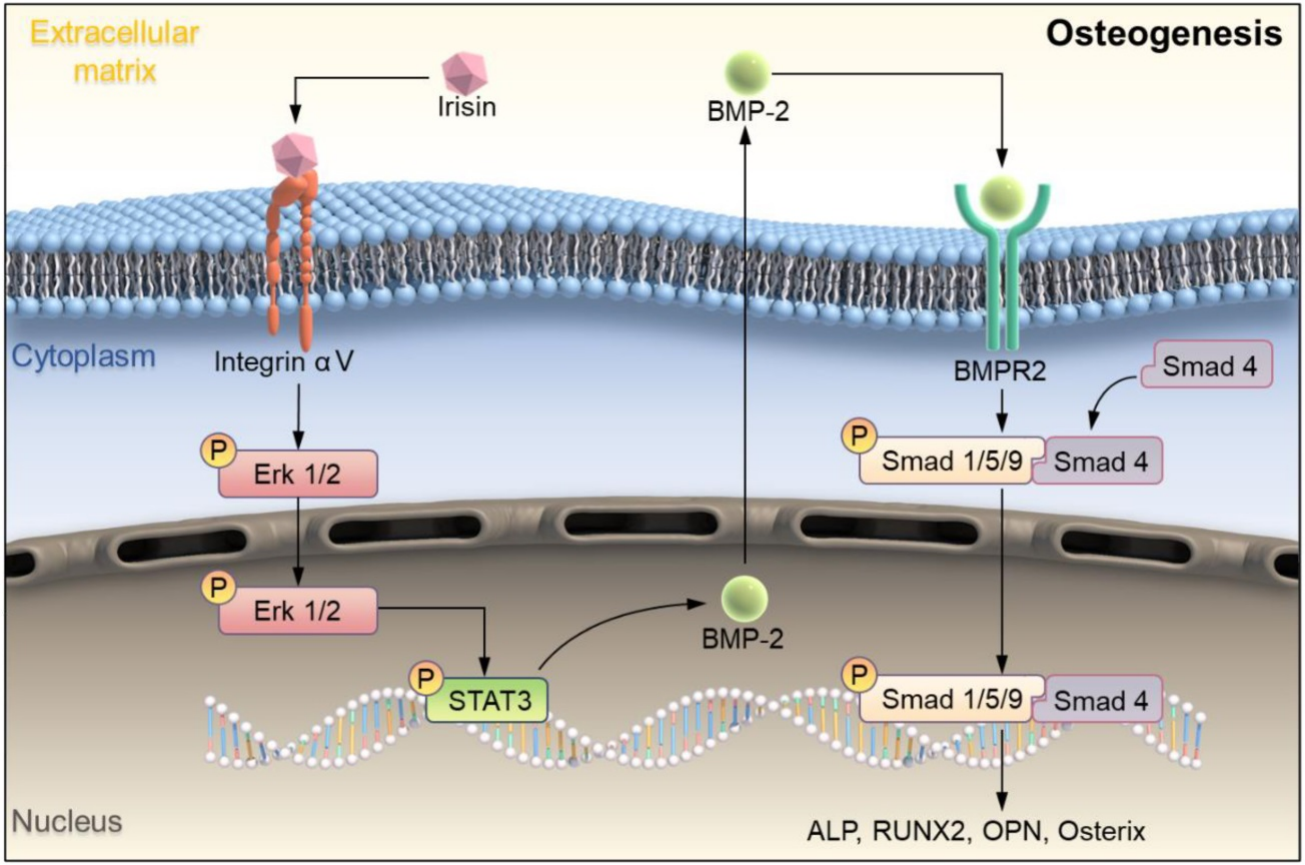
Schematic illustration indicating FNDC5-deficient mice experienced bone loss by inhibiting osteogenic differentiation and promoting osteoclast activation. (A) Schematic diagram of the mechanism of irisin regulating bone formation. Irisin combined with its receptor integrin αV on the surface of BMSCs, then in turn activated the phosphorylation of ERK and STAT3, promotes an increase of BMP2. It banded to the receptor BMPR2 on the membrane surface to activate the phosphorylation of Smad1/5/9 and promote osteogenic differentiation.

In this study, we explored the bone metabolism of irisin with FNDC5^-/-^ mice systemically. Firstly, the bone mass of FNDC5^-/-^ and WT mice at different weeks of age was analyzed by micro-CT. During the growth process of FNDC5^-/-^ mice, bone mass decreased more sharply compared with normal age-matched mice, which accelerated the process of osteoporosis. It was proved that irisin participated in the bone metabolism process *in vivo*, corresponding to the previously study 25. *In vitro* study r-Irisin not only activated ERK/STAT signaling pathway and up-regulated BMP2 expression through integrin receptor αV to promoted osteoblast differentiation from MSCs, but also had a certain inhibitory effect on osteoclast activation (Scheme 1). Our results confirmed that irisin affect both bone formation and bone resorption, and further explored the molecular mechanism of osteogenic differentiation. The results provided new ideas and directions for the research on the occurrence and development of osteoporosis.

Bone metabolic diseases include osteoporosis, end secretory bone disease, renal bone disease, hereditary bone disease and so on, among which osteoporosis is the most common disease 30, 31. Nowadays, with the rapid acceleration of aging, the incidence of osteoporosis is on the rise. Exercise can delay osteoporosis to some extent by maintaining the bone mass and strength, which is an important anti-aging factor for holding bone integrity 32. As a motion-induced muscle secretion factor, irisin is produced by the cleavage of its precursor FNDC5, which has a wide range of biological functions. In recent years, it has provided that irisin promotes the proliferation and differentiation of osteoblasts through activation of P38 and ERK pathways 21. *In vivo* and *vitro* studies on FNDC5 gene global knockout mice, Kim et al. found that irisin directly act on the integrin receptor αV on osteocytes and increased bone resorptive by adding the expression of sclerostin in osteoclast induction 24. Another *vitro* study argued that r-irisin increased the differentiation of bone marrow progenitor cells into osteoclasts in C57BL/6 mice, and positively correlated with the concentration. This increase was blocked by the integrin αVβ5 antibody, demonstrating that the integrin receptor αV may be a key factor in the regulation of osteocyte metabolism by irisin. We used FNDC5 knockout mice, and the horizontal comparison with same weeks of WT littermates, through the analysis and study of the change process of bone mass, it was found that the mice without the precursor of irisin, FNDC5, began to show significant bone loss at 12 weeks, and their BMSCs showed low osteogenic ability, while high osteoclastic activation ability of BMMs, but could be rescued by adding r-irisin. From our *vitro* studies, we confirmed that r-irisin promoted osteogenic differentiation of BMSCs and inhibited osteoclastic activation of BMMs at physiological dose 28. To sum up, we conducted a time-dimension study on the occurrence and development of osteoporosis and verified the character of irisin in bone metabolism.

Studies have shown that the main pathways which regulated bone formation including Wnt β-catenin, Hedgehog, Notch, and so on 33, 34. While, irisin could promote the proliferation and differentiation of osteoblasts by activating the P38 and ERK pathways 35. STAT3 is a transcription factor that is phosphorylated in response to various cytokines such as LIF, EGF, IL-6, OSM, and BMP2. p-STAT3 promotes RUNX2 expression by directly binding to the promoter region of RUNX220, leading to osteogenic differentiation. Osteocyte specific STAT3 deficiency reduces body weight and bone mass, and leads to spinal deformity, suggesting that STAT3 plays an important role in bone development. Aiming to explore more potential mechanisms about irisin regulates osteoblast differentiation, combined with reported studies, we found that r-irisin activated ERK/STAT pathway through binding to its receptor integrin αV and promoted the expression of BMP2. BMP is a large family of signal transduction molecules that regulates a variety of key processes, including morphogenesis, proliferation, differentiation, and apoptosis. Subsequently, Smad1/5/9 is phosphorylated and forms a complex with Smad4 and thereby allowing them to translocate into the nucleus. It regulates the expression of target proteins such as ALP, RUNX2, Osterix, OPN, promote osteogenic differentiation and eventually leads to improve bone mass, strength, and structure by promoting osteogenic differentiation 36, 37. In addition, there was an interesting finding that low BMP expression of BMSCs tended to favor adipogenic differentiation, and high expression tended to osteogenesis, which may explain why the reduction of BMP2 caused by FNDC5 deficiency would result in decreasing osteogenic ability 13. Our experimental results also proved that during the process of osteogenic differentiation of BMSCs, irisin could up-regulate the expressions of BMP2 and promote phosphorylation of Smad1/5/9, which was low expressed in bone tissues of FNDC5-deficient mice. These results revealed a new regulatory mechanism that irisin activated ERK/STAT phosphorylation through integrin receptor αV, promoted the expression of BMP2, bound with membrane receptor BMPR2, and regulated bone formation and development through BMP/SMAD signaling pathway.

In addition, osteoclast is another important part of bone remodeling and dominates bone resorption. Studies have shown that FNDC5/irisin deficient mice showed an increasing of osteoclastogenesis 38. Ma et al. found that irisin promoted the proliferation of osteoclastogenesis precursor cells, but inhibited the NF-κB signaling pathway, it activated the p38 and JNK signaling pathways, and thus inhibited the activation of osteoclasts 35. Therefore, we have also verified the increase of osteoclasts in FNDC5^-/-^ mice, and recombinant irisin could inhibit this activation. However, whether the mechanism of action is consistent with the reported mechanism still needs further investigation.

Certainly, there are still a few inevitable limitations in our study. Firstly, due to the whole gene knockout of FNDC5, which may affect the physiological activities of nerve, skeletal muscle, adipose, and other multiple tissues 39. Studies have shown that overexpression of FNDC5 promoted neural differentiation of mouse embryonic stem cells (ESCs), while FNDC5 knockout could significantly reduce the maturation of neurons and astrocytes by inhibiting neural differentiation of ESCs 40, 41. Other studies have suggested that irisin could be a myotrophic factor that induced skeletal muscle hypertrophy and rescue denervation-induced muscular atrophy 42. In brain, irisin rescinded synaptic plasticity and memory deficits in the model of Alzheimer 43. The human body is a combination of multiple organs and systems. Organs and tissues are interrelated and interacted with each other to maintain the entirety homeostasis during the process of embryo, growth, and aging. Therefore, deletion of irisin precursor FNDC5 in any organ may also cause abnormal bone metabolism. In subsequent studies, osteocyte-specific knockout of FNDC5 will be carried out for more precise exploration, especially in the study of bone metabolism mechanism.

Since the discovery of irisin in skeletal muscle, it has not only played an important role in lipid metabolism, liver, Alzheimer's disease, etc., but also in bone 43, 44. Irisin was negatively associated with sclerosing protein levels in adults with prediabetes and with vertebral fragility fractures in postmenopausal women 45. In addition, it was observed to be positively correlated with BMD at different anatomical sites in athletes 45. Some articles have shown a positive correlation between serum irisin and bone status in healthy children, and a stronger determinant of bone mineral status, rather than bone alkaline phosphatase 45. In children with type 1 diabetes, serum irisin concentration was positively correlated with bone mass and glycemic control after continuous subcutaneous insulin infusion 46.

Therefore, we advocate that exercise promotes the secretion of irisin and rescue osteoporosis. In bone defect and other diseases, exogenous irisin may be injected for bone repair. In conclusion, irisin has a critical effect on bone metabolism and provides new ideas and targets for the treatment of osteoporosis.

## Supplementary Material

Supplementary figures.

## Figures and Tables

**Table 1:** Primer sequences of mouse for qRT-PCR.

Genes	Primer sequences	
*ALP*	Forward	CTTGCTGGTGGAAGGAGGCAGG
	Reverse	CACGTCTTCTCCACCGTGGGTC
*OPN*	Forward	GCGAGGAGTTGAATGGTG
	Reverse	CTTGTGGCTGTGGGTTTC
*RUNX2*	Forward	CAAGAAGGCTCTGGCGTTTA
	Reverse	TGCAGCCTTAAATGACTCGG
*Osterix*	Forward	ATGGCGTCCTCTCTGCTTG
	Reverse	TGAAAGGTCAGCGTATGGCTT
*DC-STAMP*	Forward	TACGTGGAGAGAAGCAAGGAA
	Reverse	ACACTGAGACGTGGTTTAGGAAT
*NFATc1*	Forward	TGGGAGATGGAAGCAAAGAC
	Reverse	ATAGAAACTGACTTGGACGGG
*TRAP*	Forward	AGACCCAATGCCACCC
	Reverse	GGACCTCCAAGTTCTTATC
*OSCAR*	Forward	GTTTGGGGCTGGCAGGAATGGT
	Reverse	GAGGTGGGGAGCCGGAAATAAGG
*GAPDH*	Forward	GGTGAAGGTCGGTGTGAACG
	Reverse	CTCGCTCCTGGAAGATGGTG
