# Supplementary material for: Myokine Irisin promotes osteogenesis by activating BMP/SMAD signaling via αV integrin and regulates bone mass in mice
Source: Int J Biol Sci. 2022 Jan 1;18(2):572–84. doi: 10.7150/ijbs.63505 (PMC8741853; doi:10.7150/ijbs.63505)
Supplement: Supplementary file 1 — Supplementary figures. [file ijbsv18p0572s1.pdf]

Myokine Irisin promotes osteogenesis by activating BMP/SMAD signaling via αV integrin

Yuan Xue[1,2 #], Sihan Hu[1,2 #], Chichi Chen[1], Jiachen He[1], Jie Sun[1], Yesheng Jin[2], Yuanshu Zhang[2], Guoqing Zhu[3] *, Qin Shi[1] *, Yongjun Rui[2] *

1. Department of Orthopedics, the First Affiliated Hospital of Soochow University, Orthopedics Institute of Soochow University, Medical College of Soochow University, Suzhou, Jiangsu, 215006, P. R. China.
2. Department of Orthopedics, Wuxi Ninth People's Hospital affiliated to Soochow University, Wuxi, Jiangsu, 214026, P. R. China.
3. Department of Physiology, Nanjing Medical University, 101 Longmian Avenue, Nanjing, Jiangsu, 211166, P. R. China

# These authors contributed equally to this work.

*Corresponding author. E-mail: gqzhucn@njmu.edu.cn for Dr. Guoqing Zhu (G. Zhu), shiqin@suda.edu.cn for Dr. Qin Shi (Q. Shi), ruiyj@hotmail.com for Dr. Yongjun Rui (Y. Rui)

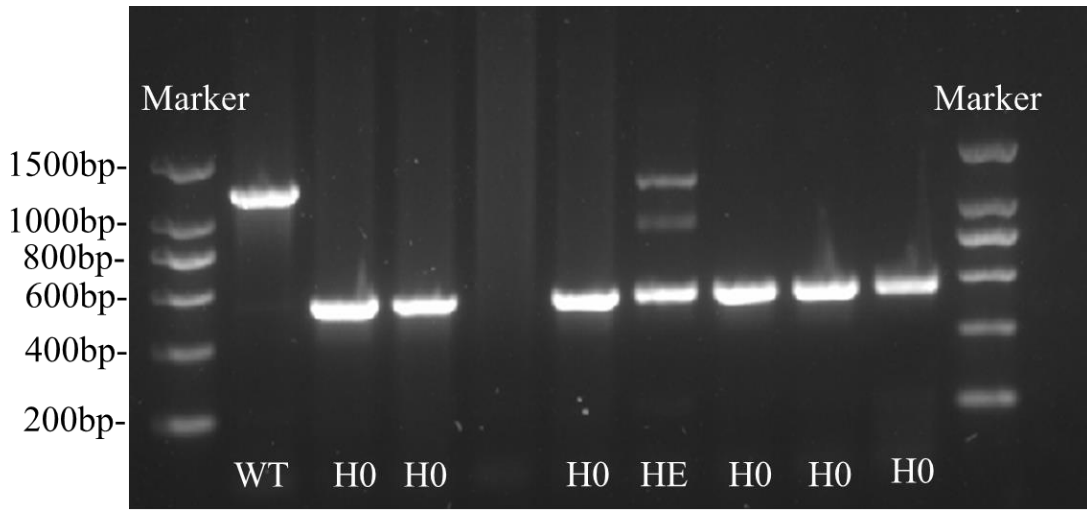

Supplementary Figure 1: Genotype identification of FNDC5$^{-/-}$ mice. HO stood for homozygous, 481bp; WT stood for wild type,1210bp; HE stood for heterozygote, 481bp&1210bp.

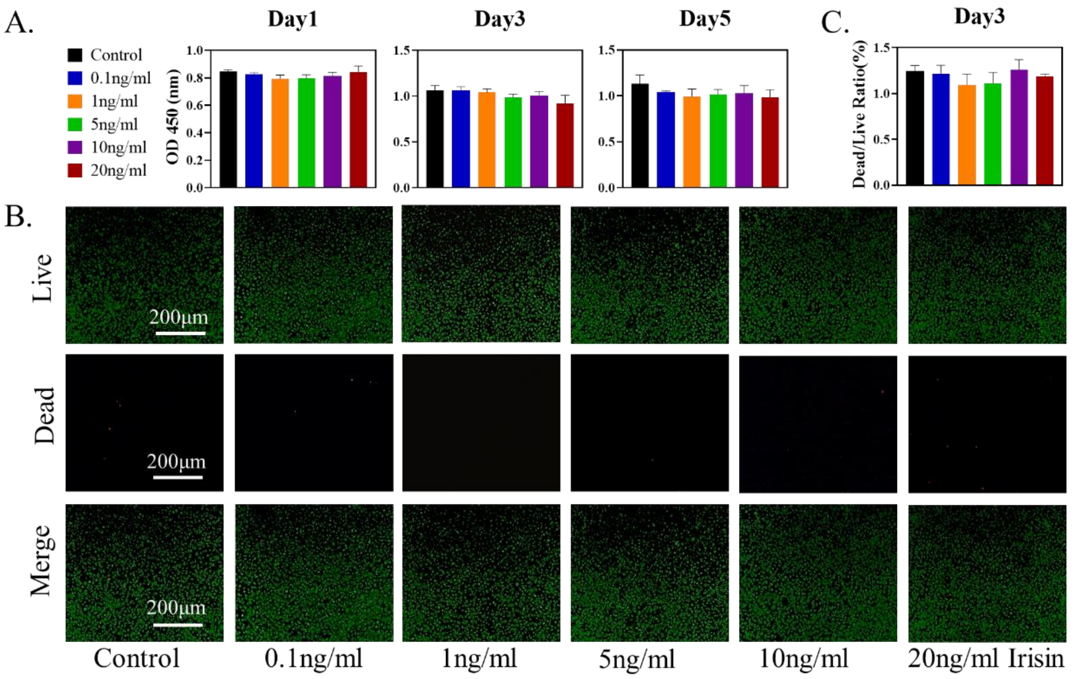

Supplementary Figure 2: Effects of different concentrations of r-irisin on the proliferation of BMSCs. (A)BMSCs were cultured under the intervention of 0.1, 1, 5, 10, 20ng/mL r-irisin, respectively, and cell viability was measured by CCK-8 on the 1st, 3rd, and 5th days. (B)Live/Dead staining on the 3rd day.

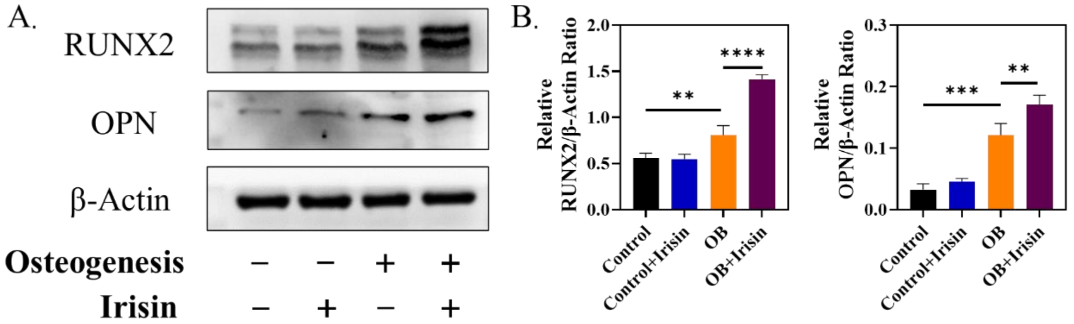

Supplementary Figure 3: (A) Western blot of OPN and RUNX2 after the intervention of recombinant irisin on osteogenic induction of BMSCs in WT mice for 7 days. (B) Quantitative analysis of Western blot. (n=5, ***P* <0.01, ****P* <0.001, *****P* <0.0001).

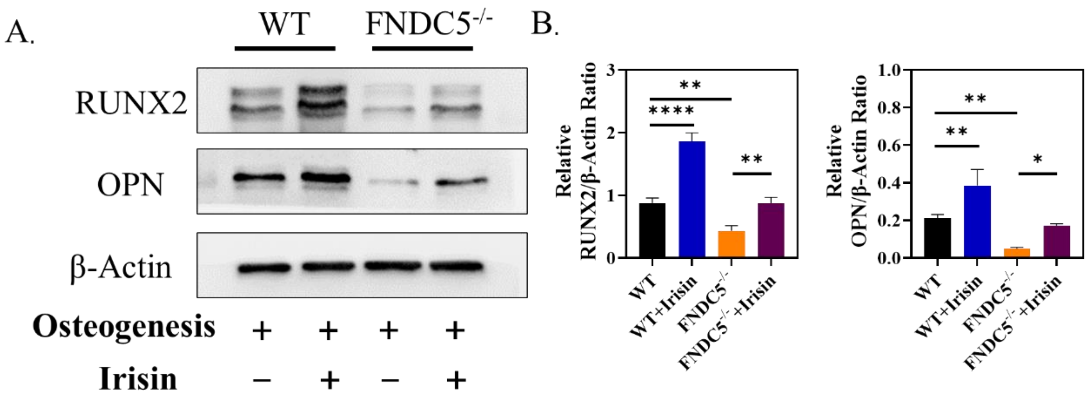

Supplementary Figure 4: Western blot analysis of RUNX2 and OPN after osteogenic induction for 7 days in BMSCs of WT and FNDC5$^{-/-}$ mice. (B) Quantitative analysis of Western blot. (n=5, **P* <0.05, ***P* <0.01, *****P* <0.0001).

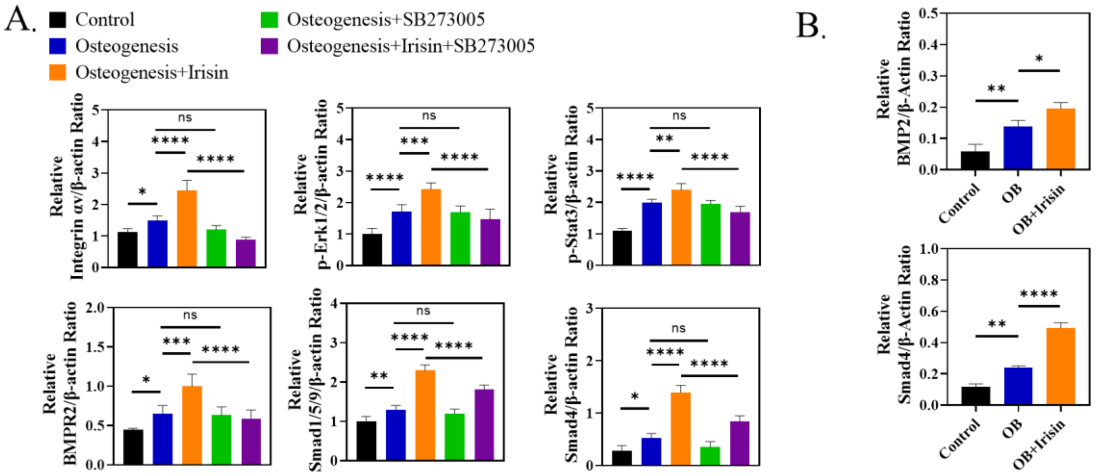

Supplementary Figure 5:(A) Quantitative analysis of WB of Protein expression levels of integrin αV, p-Erk1/2, p-STAT3, BMPR2, p-Smad1/5/9, and Smad4 of BMSCs induced into osteoblasts for 3 days in the presence of r-irisin and SB273005. (B) Quantitative analysis of WB of Protein expression levels of BMP2 and Smad4 after 3 days of osteogenic induction of BMSCs treated with r-irisin. (n=5, **P* <0.05, ***P* <0.01, ****P* <0.001, *****P* <0.0001).
